# Informatics and machine learning methods for health applications

**DOI:** 10.1186/s12911-020-01344-2

**Published:** 2020-12-30

**Authors:** Li Shen, Xinghua Shi, Zhongming Zhao, Kai Wang

**Affiliations:** 1 Department of Biostatistics, Epidemiology and Informatics, Perelman School of Medicine, University of Pennsylvania, Philadelphia, PA 19104 USA; 2 Department of Computer and Information Sciences, College of Science and Technology, Temple University, Philadelphia, PA 19122 USA; 3 Center for Precision Health, School of Biomedical Informatics, The University of Texas Health Science Center at Houston, Houston, TX 77030 USA; 4 Raymond G. Perelman Center for Cellular and Molecular Therapeutics, Childrens Hospital of Philadelphia, Philadelphia, PA 19104 USA

## Abstract

The 2020 International Conference on Intelligent Biology and Medicine (ICIBM 2020) provided a multidisciplinary forum for computational scientists and experimental biologists to share recent advances on all aspects of intelligent computing, informatics and data science in biology and medicine. ICIBM 2020 was held as a virtual conference on August 9–10, 2020, including four live sessions with forty-one oral presentations over video conferencing. In this special issue, ten high-quality manuscripts were selected after peer-review from seventy-five submissions to represent the medical informatics and decision making aspect of the conference. In this editorial, we briefly summarize these ten selected manuscripts.

## Introduction

The 2020 International Conference on Intelligent Biology and Medicine (ICIBM 2020) provided a multidisciplinary forum for computational scientists and experimental biologists to share recent advances on all aspects of intelligent computing, informatics and data science in biology and medicine. It was organized and hosted by the International Association for Intelligent Biology and Medicine (IAIBM), the University of Pennsylvania, and the Temple University on August 9–10, 2020. The conference was originally scheduled to be located in Philadelphia and was eventually transformed into a virtual conference held online due to the COVID-19 pandemic. The conference received seventy-five full-length original manuscript submissions. Each manuscript went through a rigorous review process and was peer-reviewed by at least three technical program committee members. Forty-one submissions were accepted and presented in four live sessions over Zoom, and the conference attracted ~ 300 attendees. In this special issue, ten high-quality manuscripts were selected to represent the *medical informatics and decision making* aspect of the conference. These ten articles went through the second round of peer-review and revision in the BMC Supplement submission system before their final acceptance to this ICIBM 2020 supplement issue. Of note, eight articles [1–8] are included here (Part I of the special issue), while the other two articles [9, 10] will be included in the Part II of the special issue scheduled to appear in 2021. Below we briefly summarize these ten selected manuscripts.

## Summaries of manuscripts in this issue

In “*Predicting mortality in critically ill patients with diabetes using machine learning and clinical notes*”, Ye et al. [1] investigated the mortality rate of patients with diabetes admitted to intensive care unit (ICU), using machine learning and clinical notes, while previous work was mostly based on regression models and did not take clinical notes into considerations. Specifically, the authors proposed to use Unified Medical Language System (UMLS) resources, coupled with machine learning and natural language processing (NLP) approaches, to predict the risk of mortality. They implemented rule-based feature engineering and knowledge-guided deep learning approaches and trained a knowledge-guided convolutional neural network (CNN) model with word embeddings and UMLS Concept Unique Identifier (CUI) entity embeddings. The comparative study with a few competing methods was performed on the data from Medical Information Mart for Intensive Care III (MIMIC-III) and demonstrated the promise of the proposed method. The results indicate that machine learning models along with NLP of clinical notes provide powerful tools to help doctors predict mortality in critically ill diabetic patients.

In “*Natural Language Processing (NLP) tools in extracting biomedical concepts from research articles: a case study on autism spectrum disorder*”, Peng et al. [2] performed a comparative study of NLP tools in extracting biomedical concepts from research articles. This study compared three NLP software tools CLAMP, cTAKES, and MetaMap, which are among the most widely used tools in the field. Autism spectrum disorder (ASD) was used as a case study for the comparison. In the test bed, the authors collected 821 ASD-related terms from the literature as the benchmark for evaluation, and then extracted ASD-specific vocabulary from 544 full-text articles and 20,408 abstracts of PubMed and followed by semantic type filtering. For F1 measure, CLAMP performed the best and followed by cTAKES and then MetaMap. In addition, CLAMP yielded much higher precision than cTAKES and MetaMap, while cTAKES and MetaMap had higher recall than CLAMP. The findings provide valuable guideline for future application of these tools.

In “*Stress detection using deep neural networks*”, Li et al. [3] applied deep neural network models to predict/detect stress from wearable sensor signals. The authors implemented a deep 1D convolutional neural network model and a deep multilayer perceptron neural network model. Both models were applied to analyze physiological signals measured from chest-worn and wrist-worn sensors in order to perform two classification tasks. The two classification tasks are (1) binary stress detection and (2) 3-class emotion classification. Comparative studies with traditional machine learning methods were performed empirically. The proposed deep learning models yielded highly promising predication accuracy.

In “*Annotation and extraction of age and temporally-related events from clinical histories*”, Hong et al. [4] reported new development of an annotated corpus to facilitate information extraction from clinical notes, specifically with the focus on age and temporally-related events in clinical history. First, the authors expanded the ShARe Semantic Schema to support the representation of age, temporal and family history information. Second, they applied this annotation schema on the 2014 ShARe eHealth Challenge corpus, and captured valuable new information under the new age and temporal information classes. Third, they developed a prototype rule-based NLP system to extract clinical events with age and temporal mentions, which yielded promising results. An interesting future direction is to develop a hybrid rule-based and deep learning NLP system. In short, the proposed annotation schema and NLP system is able to encode historical events from clinical notes, which is expected to provide valuable information to support clinical and translational research studies.

In “*SURF: identifying and allocating resources during out-of-hospital cardiac arrest*”, Rao et al. [5] studied a significant clinical problem, which is to optimize the resource allocation in the public setting for treating patients with sudden cardiac arrest. They presented a conceptual framework to analyze the needs for rapid response and the involved users as well as their workflow. To match the user and the Automated External Debrillators (AEDs), they used Bipartite Matching and Integer Linear Programming. Given the high time complexity of these two methods, they further proposed a new method called Preprocessed Integer Linear Programming. Empirical studies were performed on the simulated data, and demonstrated greatly improved efficiency of the proposed method, which indicated its potential to allow matching of users with AEDs in real-time during cardiac emergency.

In “Utilizing deep learning and graph mining to identify drug use on twitter data”, Tassone et al. [6] proposed a deep learning method based on CNN to identify tweets about drug use. The authors trained two CNN-based classifiers. The first one used 2,661 manually labeled samples, and the second one included synthetically generated tweets. Empirical studies were performed in comparison with a few competing methods including support vector machine (SVM), XGBoost, and Bidirectional Encoder Representations from Transformers (BERT), and the authors demonstrated a significant improvement using CNN-based models. Combining association rule mining with the CNN-based classifier identified keywords such as “smoke", “cocaine", and "marijuana" that triggered a drug-positive classification. The proposed method is expected to help address the important problem of analyzing drug use of patients using social media data.

In “*An interpretable risk prediction model for healthcare with pattern attention*”, Kamel et al. [7] developed an interpretable deep learning model to predict the patients' outcomes based on the medical records data. This model is named as Pattern Attention model with Value Embedding (PAVE), which has the following strengths. First, it takes into account real-value medical events by embedding the values into vectors, without needing to impute the missing values. Second, based on attention mechanisms, PAVE can use the attention weights to interpret the model's clinical outputs at both the event pattern level and the single event level. Extensive empirical studies on two real-world electronic health record (EHR) datasets demonstrated the improved prediction performance of PAVE over the state-of-the-art competing methods, as well as its capability for detection of many medical event patterns with high contribution rates to mortality and sepsis onset. In short, PAVE provides a promising method for interpretable clinical risk prediction from the EHR data.

In “*Comparing different wavelet transform on removing electrocardiogram baseline wanders and special trends*”, Chen et al. [8] addressed a signal processing problem of Electrocardiogram (ECG), an important tool used in clinical applications of cardiovascular disorders. ECG signals are often corrupted by low-frequency baseline wander (BW) artifact, which may lead to faulty interpretations. Wavelet transform has been shown to be among the most effective method for BW removals. This paper went one step further and performed a comparative study of different wavelets in their ability to remove the baseline errors and preserve the original signal. Daubechies-3 and Sym-3 wavelets were found to have the best performance. These findings could facilitate future real-time processing of streaming ECG signals for clinical decision support systems.

In “*Unsupervised phenotyping of sepsis using non-negative matrix factorization on temporal trends from a multivariate panel of physiological measurements*”, Ding et al. [9] performed a study of unsupervised clustering of patients with sepsis admitted to the ICU, and the goal is to identify novel clinical phenotypes and to inform targeted therapies and improved care. They employed Subgraph-Augmented Non-negative Matrix Factorization (SANMF) and frequent subgraph mining to derive clinically relevant sepsis phenotypes from temporal trends of physiological data. The study was performed on the MIMIC-III data, including a cohort of 5,782 patients. Three novel phenotypes were identified to have distinct clinical characteristics and independent association with patient outcomes. These findings can help understand the disease heterogeneity and guide further treatment planning.

In “*Identifying risk factors of preterm birth and perinatal mortality using statistical and machine learning approaches*”, Kothiya et al. [10] studied the problem of how maternal health and lifestyle affect pregnancy outcomes. They applied statistical and machine learning methods to the medical record and life style data for predicting preterm birth and perinatal mortality. A data set with 570 variables from 3,122 enrolled women were analyzed to predict three birth outcomes: preterm living infant, full-term living infant, or perinatal mortality. Random forest was used for feature selection, followed by correlation analysis and logistic regression analysis for relating medical record and life style features to the birth outcome. These analyses identified several significant associations between maternal factors in the electronic health record data and pregnancy outcome.

## Discussion

Most of the studies included here were facilitated by and conducted with the valuable data resources either available in the open science domain or accessible to the authors. In five of these articles [1, 4, 7, 9, 10], electronic health record (EHR) data were analyzed for various purposes, including (1) predicting outcomes such as mortality [1, 7, 10], sepsis [7], and preterm birth [10], (2) annotation and extraction of age and temporally-related events [4], and (3) sepsis phenotyping [9]. In the remaining five articles [2, 3, 5, 6, 8], the authors analyzed (1) publication data for extracting biomedical concepts [2], (2) wearable sensor data for stress detection [3], (3) location data for resource allocation for cardiac emergency [5], (4) social media data for drug use detection [6], and (5) ECG data for biomedical signal denoising [8].

In these studies, the authors employed informatics and machine learning methods to address various health topics, including diabetes [1], autism spectrum disorder [2], stress [3], health research in general [4], cardiac arrest [5], drug use [6], sepsis [7, 9], heart disorders [8], and preterm birth and perinatal mortality [10]. To address the biomedical problems in the above health applications, these studies employed a wide range of informatics and machine learning methods, including deep learning [1, 3, 6, 7], NLP [1, 2, 4], matching algorithms [5], association mining [6], wavelet analysis [8], factor analysis [9], frequent graph mining [9], and traditional statistical machine learning [10].

In summary, we envision a growing interest of medical informatics and machine learning approaches to address the pressing problems in health applications. We anticipate that future ICIBM events will continue serving as a forum for researchers to exchange ideas, data, and software, and speed up the development of intelligent computing methods for data-driven discovery in biology and medicine.
